# Molecular characteristics and prognostic insights into *BRCA*-associated breast cancer in Kazakhstan

**DOI:** 10.1038/s41598-026-36086-0

**Published:** 2026-01-17

**Authors:** Ainur Samigatova, Nursulu Altaeva, Yerlan Suleimenov, Petr Sibiryakov, Kuantkan Zhabagin, Zhanar Zhakypbekkyzy, Bakhytzhan Seksenbayev, Noso Yoshihiro, Oxana Tsigengagel

**Affiliations:** 1 Astana Medical University, QazGene LLP, Beibitshilik Street 49/A, Astana, 010000 Kazakhstan; 2 Department of Medical Genetics and Molecular Biology, Astana Medical University, Astana, Kazakhstan; 3 CDL OLYMP- LLP, QazGene LLP, Astana, Kazakhstan; 4 GeneNote LLP, Almaty, Kazakhstan; 5 Medical Center Hospital of The President’s Affairs Administration of The Republic of Kazakhstan, Astana, Kazakhstan; 6 Astana Medical University, Astana, Kazakhstan; 7 Hiroshima International University, Kurose gakuendai, Higashi hiroshima, Hiroshima, Japan; 8 Department of Epidemiology and Biostatistics, Astana Medical University, Astana, Kazakhstan

**Keywords:** Breast cancer, *BRCA1*/*BRCA2* mutations, Molecular profiling, Clinical outcomes, Kazakhstani population, Cancer, Genetics, Oncology

## Abstract

Breast cancer remains the leading cause of cancer morbidity and mortality among women worldwide. Approximately 5–10% of cases involve pathogenic *BRCA1/2* variants, typically associated with early-onset, aggressive disease. This study aimed to determine the frequency and spectrum of *BRCA1/2* mutations among Kazakhstani breast cancer patients and to analyze their associations with clinicopathological features and survival outcomes. A total of 186 patients aged 21–90 years were examined between December 2023 and June 2024 using next-generation sequencing and retrospective chart review. The median age was 44 years for *BRCA1*, 48 for *BRCA2*, and 51 for *BRCA*-negative patients. Advanced disease (stages III-IV) was more common among mutation carriers (*p* < 0.001). Pathogenic *BRCA1* and *BRCA2* variants were found in 22% and 9% of cases, respectively; *BRCA1* tumors were predominantly triple-negative (88%), whereas *BRCA2* tumors were mainly hormone receptor–positive (65%). Both subtypes were largely poorly differentiated (61% and 53%, respectively). The median progression-free survival was 34, 12, and 8 months for *BRCA*-negative, *BRCA1*-, and *BRCA2*-positive groups, respectively (*p* = 0.001). To our knowledge, this is the first comprehensive analysis of *BRCA1/2* mutation profiles among Kazakhstani women, highlighting distinct genotype-phenotype correlations and supporting the need for personalized therapeutic strategies in this population.

**Supplementary Information:**

The online version contains supplementary material available at 10.1038/s41598-026-36086-0.

## Introduction

Breast cancer (*BC*) remains one of the most significant medical and social challenges of modern healthcare, consistently ranking among the leading causes of cancer incidence and mortality in women worldwide. According to the *Global Cancer Observatory (GLOBOCAN*,* 2022*), over 2.3 million new cases of *BC* are diagnosed annually, accounting for 11.6% of all malignancies, with 665,684 deaths attributed to the disease worldwide. By incidence, it is second only to lung cancer (12.4%) and ahead of colorectal cancer (9.6%), while in terms of mortality, it ranks fourth globally (6.9%), following lung (18.7%), colorectal (9.3%), and liver cancer (7.8%)^1^.

In Kazakhstan, the burden of breast cancer remains persistently high, with over 5,500 new cases and more than 1,000 related deaths registered in 2023. In the same year, *BC* accounted for 14.9% of all malignant neoplasms, compared with 14.7% in 2022, indicating a stable yet unfavorable epidemiological trend. In mortality structure, *BC* ranks third (8.1%), following lung (15.8%) and gastric cancer (11.9%), which exceed the global average mortality rate for this malignancy^2^. These data underscore the urgent need to investigate the biological and molecular characteristics of *BC* within the Kazakhstani population, accounting for regional, ethnogenetic, and molecular diversity.

Hereditary predisposition, particularly *BRCA1/2* mutations, is a significant determinant of breast cancer biology. Although 25–30% of cases have a hereditary component, only 5–10% involve germline mutations, with *BRCA1/2* conferring over a fourfold increase in lifetime risk and raising cumulative risk to nearly 80%^3,4^. Beyond genetic susceptibility, *BRCA1/2*-associated tumors show marked genomic instability, structural *DNA* alterations, and global hypomethylation, distinguishing them from sporadic forms^5^. *BRCA1* tumors are typically triple-negative, poorly differentiated, and highly proliferative, whereas *BRCA2* tumors more often display luminal characteristics and endocrine responsiveness, though some may behave aggressively^6–9^. These molecular and phenotypic distinctions underscore the need for region-specific studies, as the frequency and expression of *BRCA* mutations vary considerably among populations.

Despite extensive international research, data on the prevalence and clinicopathological characteristics of *BRCA1/2* mutations in Central Asia, including Kazakhstan, remain scarce. A systematic review comprising 70 studies and 17,872 cases reported that *BRCA1/2* mutation frequencies varied widely - from 1.8% to 36.9% - depending on geography, inclusion criteria, and molecular subtype^10^. For instance, in Japan, *BRCA1/2* mutations were identified in 20.0% of triple-negative *BC* cases, increasing to 41.4% among those with a positive family history^11^. In Turkey, *BRCA1* and *BRCA2* mutations were detected in 7.8% and 5.4% of patients, respectively, with significantly earlier onset of disease among mutation carriers (39.7 and 41.1 vs. 43.2 years; *p* < 0.001), particularly in the triple-negative subgroup^12^. However, no systematic studies have been conducted in Kazakhstan to evaluate the relationship between *BRCA* status, age at onset, tumor stage, molecular and morphological patterns, hormonal profiles, metastatic patterns, and survival outcomes.

International evidence suggests that *BRCA1* mutations are generally associated with more aggressive disease, early metastatic spread, and poorer survival outcomes^13,14^. Conversely, *BRCA2* mutations are more frequent in hormone receptor–positive subtypes, yet may also contribute to unfavorable prognoses under certain conditions^15^. Moreover, *BRCA*-deficient tumors demonstrate heightened sensitivity to *DNA*-damaging agents, including *PARP* inhibitors and platinum-based chemotherapy, but may exhibit resistance to endocrine therapy in some *HR*⁺ cases^16^. These biological distinctions emphasize the importance of studying *BRCA*-associated breast cancer within diverse populations, as genetic background and environmental factors may substantially influence mutation prevalence and clinical manifestations.

Consistent with these biological observations, findings from the large multicenter *ESME* cohort (2008–2016, *n* = 20,624) revealed that the prognostic impact of *BRCA1/2* mutations varies by molecular subtype. In the triple-negative subgroup, *BRCA* mutations were associated with improved survival outcomes (*OS: HR* = 0.76; *PFS: HR* = 0.69), whereas among *HR*^+^/*HER2*^-^ patients, decreased progression-free survival (HR = 1.23; *p* = 0.024) and a trend toward reduced overall survival were observed^17^. These results further highlight the heterogeneity of *BRCA*-associated breast cancers and underscore the need for molecularly guided, personalized therapeutic approaches.

Comprehensive characterization of *BRCA*-associated breast cancer in Kazakhstan is essential to address existing knowledge gaps. Defining the prevalence and spectrum of *BRCA1/2* mutations will enable the identification of high-risk women and improve precision screening and management. Analysis of clinicopathological and molecular features will refine prognostic markers and inform therapeutic strategies, including *PARP* inhibitors, platinum agents, and targeted combinations. This first large-scale study aims to elucidate the genetic and clinical landscape of *BRCA*-associated *BC* in Kazakhstan, advancing personalized oncology and contributing regional data to global research on hereditary breast cancer.

## Materials and methods

### Patients

Between December 2023 and June 2024, 186 patients aged 21 to 90 years were referred to *OLYMP*, Kazakhstan’s largest molecular diagnostic laboratory, for germline whole-genome sequencing. This was a single-center ambispective observational cohort study. The cohort did not represent an unselected consecutive series; only patients diagnosed and treated at the Astana Multidisciplinary Medical Center who met the *National Comprehensive Cancer Network (NCCN) Genetic/Familial High-Risk Assessment: Breast and Ovarian Guidelines (version 2022)*^18^ were eligible. Eligibility criteria included age at diagnosis, tumor subtype (including triple-negative breast cancer), sex, personal and/or family history of breast, ovarian, pancreatic, or aggressive prostate cancer, and the presence of known pathogenic variants in relatives.

Given the high cost and limited availability of germline whole genome sequencing in Kazakhstan, the study intentionally included both early-stage and metastatic breast cancer patients who met high-risk criteria. Early-stage cases were included to allow comparative assessment of clinical behavior and disease aggressiveness associated with *BRCA1/2* pathogenic variants, while metastatic cases were included at the time of progression to evaluate outcomes and therapy response. Patients with early-stage and metastatic breast cancer were analyzed separately due to differences in clinical course, treatment strategies, and outcome measures.

The study cohort also included patients whose primary breast cancer diagnosis had been established more than five years before the onset of metastatic disease and who had been under longitudinal follow-up during remission. Pathogenic variants in *BRCA1* or *BRCA2* were identified in 58 patients. Demographic data, family cancer history, treatment types, and tumor histopathology were collected through retrospective chart review and patient interviews, while clinical outcomes and treatment data from the time of metastatic progression were collected prospectively. All patients included in the study were managed in strict accordance with the standardized national breast cancer treatment protocols of the Republic of Kazakhstan, with therapeutic interventions determined according to disease stage and individual clinical characteristics, ensuring comprehensive adherence to national guidelines^19^.

**Fig. 1:**
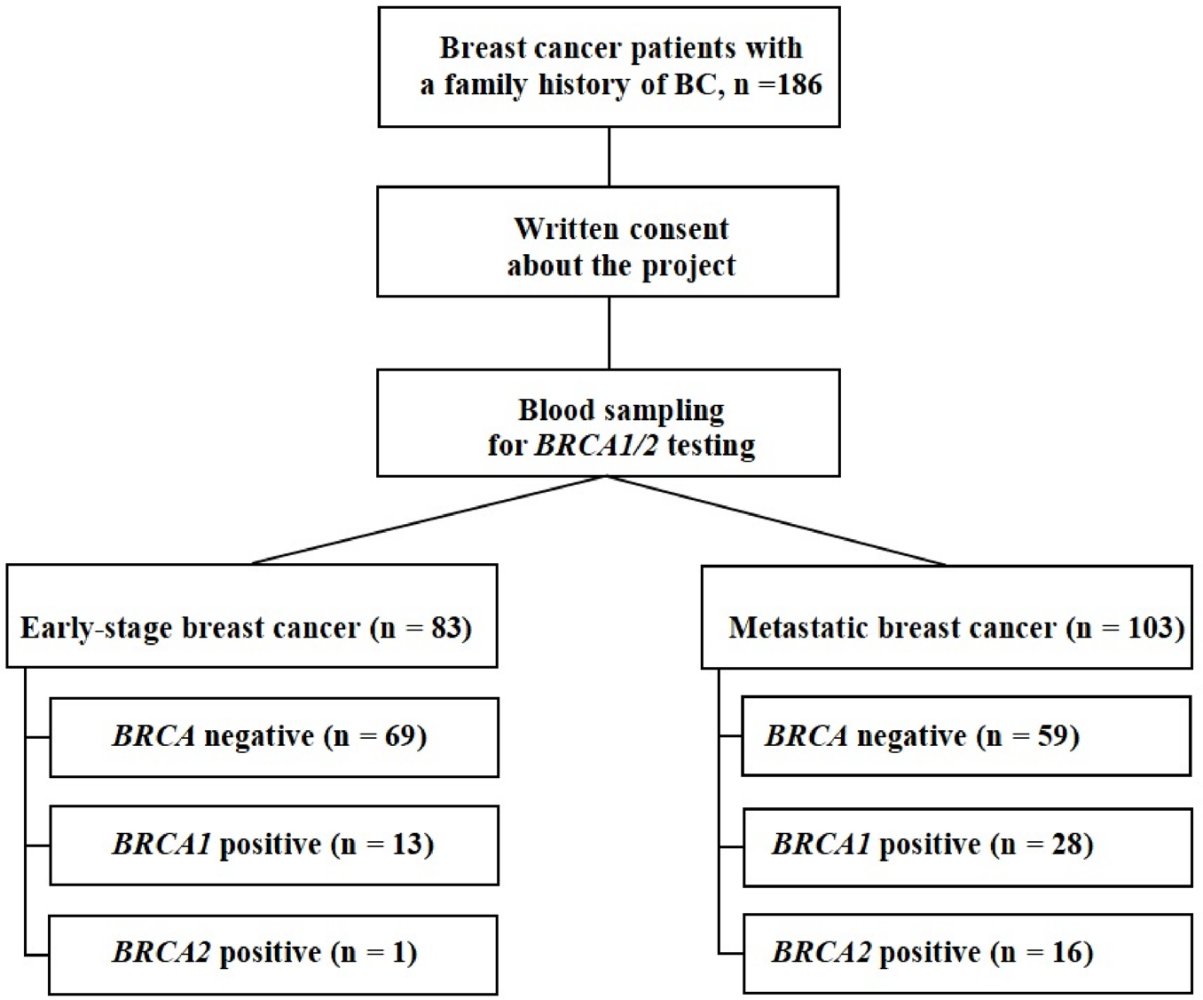
Flowchart of patient selection and *BRCA1/2* status stratified by disease setting (early vs. metastatic breast cancer).

Patients were classified according to disease setting as having early breast cancer (non-metastatic disease at diagnosis) or metastatic breast cancer (presence of distant metastases) (Fig. 1). A patient selection flowchart is provided to illustrate eligibility criteria, cohort composition, and subgroup stratification. Patients will continue to be followed longitudinally to capture subsequent disease progression and outcomes.

### Potential ethical concerns

The study was reviewed and approved by the Ethics Committee before initiation. Approval was granted by the Local Ethics Committee of Kazakhstan’s Astana Multidisciplinary Medical Center (Protocol No: IRB-A14, dated 15/06/2023). All procedures were conducted in accordance with relevant guidelines and regulatory standards. Written informed consent was obtained from all participants after they were fully informed about the study’s purpose, procedures, and their right to withdraw at any time. Confidentiality and anonymity were ensured throughout the study, in full compliance with bioethical principles and international research guidelines.

### Immunohistochemical profile: Estrogen receptor (*ER*), progesterone receptor (*PR*), human epidermal growth factor receptor 2 (*HER2*), and *Ki-67*

The expression levels of estrogen receptor (*ER*), progesterone receptor (*PR*), human epidermal growth factor receptor 2 (*HER2*), and *Ki-67* were determined from pathology reports. Negativity for *ER* and *PR* was defined as immunohistochemical (*IHC*) staining in less than 1% of tumor cells. *HER2* status was evaluated by *IHC* using a three-point scale: +1 indicated negativity, + 3 indicated positivity, and 2 + required confirmation of gene amplification by fluorescence in situ hybridization (*FISH*). *Ki-67* status was assessed by IHC and reported as the percentage of positive tumor cell nuclei. Triple-negative breast cancer (*TNBC*) was characterized by the absence of *ER* and *PR* expression and a negative *HER2* status.

### Detection of mutations in the *BRCA1/2* genes by next generation sequencing (NGS)

Identification of *BRCA1/2* gene alterations was performed exclusively using NGS. NGS is a high-throughput, highly sensitive, and specific molecular technique. It enables comprehensive detection of sequence variants across the entire coding region of the gene. For NGS, genomic DNA was extracted from peripheral venous blood samples using standardized, commercially available reagent kits. Extraction followed the manufacturer’s recommendations. The amplified libraries were evaluated qualitatively and quantitatively. Library preparation used the *“Quasar-BRCA1/2”* reagent kit (TestGen, Russia; TU 21.20.23–038.23-97638376-2020) and followed validated laboratory protocols. Sequencing was performed on the Illumina MiSeq platform (Illumina, USA). The mean sequencing depth exceeded 50×. This ensured reliable identification of heterozygous and homozygous variants with high analytical accuracy. Variant annotation and interpretation followed the *ACMG* guidelines for sequence variant classification^20^.

### Statistical analysis

Survival endpoints were defined as follows: overall survival (OS) - the time from breast cancer diagnosis to death or last follow-up; disease-free survival (DFS) - the interval from complete remission or completion of primary treatment to the first documented recurrence; and progression-free survival (*PFS*) - the time from the initiation of systemic therapy to disease progression or death, whichever occurred first. Patients alive at the end of observation were censored at their last contact. Survival distributions were estimated using the Kaplan–Meier method. Intergroup differences were assessed with the log-rank test.

Comparative analyses of clinical and tumor characteristics used the Pearson χ² test. Fisher’s exact test was applied when expected cell frequencies were < 5. Continuous variables were analyzed with the non-parametric Mann-Whitney U test. All statistical analyses were performed using SPSS Statistics version 28.0 (IBM) and SAS software version 9.3 (SAS Institute). Statistical significance was a two-sided p-value < 0.05.

## Results

### Patient characteristics and BRCA status

Among 186 patients with breast cancer, 58 carried pathogenic or likely pathogenic germline *BRCA1/2* variants (41 *BRCA1*, 17 *BRCA2*), while 128 patients had no clinically significant alterations. The median age was 51 years (range: 46–62) in *BRCA*-negative patients, 44 years^40–46^ in *BRCA1*-positive patients, and 48 years (43–54) in *BRCA2*-positive patients. Overall, 22% of patients were *BRCA1* carriers and 9% were *BRCA2* carriers.

### Disease stage and pathological characteristics

Stage distribution differed significantly between *BRCA* subgroups (*p* < 0.001). In the BRCA-negative group, most patients presented with stage II or III disease (*n* = 60 and 44, respectively). In contrast, stage III and IV disease predominated in *BRCA1*-positive patients (*n* = 17 and 10, respectively), whereas *BRCA2*-positive patients had no stage I cases and primarily stage III or IV disease. Tumor size and lymphovascular invasion did not differ significantly among groups, while axillary lymph node involvement was observed in 61% of *BRCA*-negative, 46% of *BRCA1*-positive, and 71% of *BRCA2*-positive patients (Table 1).

### Metastatic patterns

Distant metastases varied significantly across subgroups (*p* = 0.001). Among *BRCA*-negative patients, 69 (54%) had no metastases, compared with 32% in the *BRCA1*-positive group and 6% in the *BRCA2*-positive group. Lung metastases were more frequent in *BRCA1*-positive patients, while multiple organ involvement was most common in *BRCA2*-positive patients. Concurrent breast and ovarian cancers were observed predominantly in *BRCA1*-positive cases (Table 1).

### Family history

A family history of breast cancer was identified in 77 *BRCA*-negative, 6 *BRCA1*-positive, and 3 *BRCA2*-positive patients. A family history of ovarian cancer was observed in 51, 1, and 6 patients, respectively. Multiple affected relatives were documented exclusively among *BRCA*-positive patients (*n* = 34 *BRCA1*, *n* = 8 *BRCA2*) and were absent in the *BRCA*-negative cohort (Table 1).

**Table 1:** Comparison of clinical characteristics in patients with *BRCA*-negative vs. *BRCA1* vs. *BRCA2* (*n* = 186).

Variables	BRCA-negative *n* = 128 (69%)	BRCA1-positive *n* = 41 (22%)	BRCA2-positive *n* = 17 (9%)	*p* value
Age, median (IQR)	51 (46–62)	44 (41–48)	48 (43–54)	0.001
Stage, n (%)				
I	16 (13%)	5 (12%)	0	0.001
II	60 (47%)	9 (22%)	3 (18%)	
III	44 (34%)	17 (42%)	7 (41%)	
IV	8 (6%)	10 (24%)	7 (41%)	
Tumor size (%)				0.973
> 2см	49 (38%)	16 (39%)	7 (41%)	
≤ 2см	79 (62%)	25 (61%)	10 (59%)	
Status of axillary lymph nodes (%)				0.147
negative	50 (39%)	22 (54%)	5 (29%)	
positive	78 (61%)	19 (46%)	12 (71%)	
Localization of distant metastases (%)				0.001
mts negative	69 (54%)	13 (32%)	1 (6%)	
lung	10 (8%)	12 (29%)	1 (6%)	
bone	12 (9%)	5 (12%)	3 (18%)	
liver	12 (9%)	2 (5%)	0	
brain	8 (6%)	0	1 (6%)	
multiple metastasis	10 (8%)	3 (7%)	10 (58%)	
other solitary lymph nodes (lymph nodes, peritoneum, adrenal glands, kidneys, etc.)	7 (6%)	6 (15%)	1 (6%)	
Primary multiple cancer				0.010
negative	125 (97%)	32 (78%)	16 (94%)	
cancer of the second breast	1 (1%)	3 (8%)	1 (6%)	
ovarian cancer	0	3 (8%)	0	
uterine cancer	0	1 (2%)	0	
stomach cancer	0	1 (2%)	0	
vaginal cancer	0	1 (2%)	0	
thyroid cancer	1 (1%)	0	0	
colon cancer	1 (1%)	0	0	
Family history of cancer				0.001
Any breast cancer in relatives	77	6	3	
Ovarian cancer in relatives	51	1	6	
Multiple affected relatives	0	34	8	

### Tumor grade, histological subtype, proliferation, and receptor status

Tumor grade differed significantly between *BRCA* subgroups (*p* = 0.002): moderately differentiated tumors (*G*2) predominated in *BRCA*-negative patients (45%), whereas poorly differentiated tumors (*G*3) were more frequent in *BRCA1* (61%) and *BRCA2* (53%) carriers. Histological subtype distribution did not differ by *BRCA* status (*p* = 0.926). Invasive ductal carcinoma predominated in all groups (*BRCA*-negative: 108/128, 84%; *BRCA1*-positive: 35/41, 86%; *BRCA2*-positive: 14/17, 82%), followed by invasive lobular carcinoma (13/128, 10%; 5/41, 12%; 2/17, 12%, respectively). Mixed histology was rare (7/128, 6%; 1/41, 2%; 1/17, 6%). The median *Ki-67* index was 40% (IQR 20–55), higher in *BRCA1* carriers than in non-carriers (63% vs. 16%, *p* = 0.001). *BRCA1*-associated tumors showed higher proliferative activity than *BRCA2*-associated tumors, with median *Ki-67* values of 75% and 55%, respectively. Among *BRCA*-negative patients, 80% were *ER*-positive, 79% *PR*-positive, and 22% *HER2*-positive. In the *BRCA2* subgroup, 65% of tumors were *ER/PR*-positive, and *HER2* positivity was detected in 18% of cases (*n* = 3). In contrast, only 12% of *BRCA1* carriers expressed ER/PR, and none expressed *HER2*. Consequently, triple-negative tumors were more frequent in *BRCA1* than in *BRCA2* (88% vs. 29%) and *BRCA*-negative groups (88% vs. 12%; *p* = 0.001) (Table 2).

**Table 2:** Comparison of pathological characteristics in patients with *BRCA* negative vs. *BRCA1* vs. *BRCA2* (*n* = 186).

Variables	BRCA-negative *n* = 128 (69%)	BRCA1-positive *n* = 41 (22%)	BRCA2-positive *n* = 17 (9%)	*p* value
Degree of differentiation (Grade)				0,002
*G1*	25 (20%)	1 (2%)	0	
*G2*	58 (45%)	15 (37%)	8 (47%)	
*G3*	45 (35%)	25 (61%)	9 (53%)	
Histological Subtype				0,926
IDC (Invasive Ductal Carcinoma)	108 (84%)	35 (86%)	14 (82%)	
ILC (Invasive Lobular Carcinoma)	13 (10%)	5 (12%)	2 (12%)	
Mixed	7 (6%)	1 (2%)	1 (6%)	
Proliferation index (Кi-67)				
Кi-67, median (range)	40 (20–55)	75 (57–85)	55 (20–65)	0,001
Кi-67 ≤ 30%	51 (40%)	4 (10%)	7 (41%)	0,001
Кi-67 = 30–70%	57 (44%)	11 (27%)	10 (59%)	
Кi-67 ≥ 70%	20 (16%)	26 (63%)	0	
*HER2*neu status, n (%)				0,005
negative	100 (78%)	41 (100%)	14 (82%)	
positive	28 (22%)	0	3 (18%)	
Estrogen receptor status, n (%)				0,001
negative	26 (20%)	36 (88%)	6 (35%)	
positive	102 (80%)	5 (12%)	11 (65%)	
Progesterone receptor status, n (%)				0,001
negative	27 (21%)	36 (88%)	6 (35%)	
positive	101 (79%)	5 (12%)	11 (65%)	
Triple negative, n (%)				0,001
negative	106 (83%)	5 (12%)	12 (71%)	
positive	22 (17%)	36 (88%)	5 (29%)	

### *BRCA1*/2 variant spectrum

A total of 22 pathogenic or likely pathogenic variants were identified in the *BRCA1* and *BRCA2* genes, with 11 variants detected in each gene. In *BRCA1* (transcript NM_007294.4), the majority were loss-of-function alterations, including frameshift, nonsense, and splice-site variants, predominantly located in exons 10, 12, 21, and 23. The most frequent variant was c.5381_5382insC (p.Glu1794Aspfs36) in exon 21 (*n* = 12). Other recurrent variants included c.981_982del (p.Cys328), c.3214del (p.Leu1072), and c.5470_5477del (p.Ile1824Aspfs3), while the splice-site variant c.5277 + 1G > A was observed in three cases. In *BRCA2* (transcript NM_000059.4), most alterations were predicted to result in protein truncation or aberrant splicing, distributed mainly across exons 11, 16, and 24. Loss of exon 16 was detected in six patients, and recurrent frameshift variants included c.2600_2601insA (p.Thr868Tyrfs13) and c.9241_9242insA (p.Val3081Aspfs*30). Several splice-site variants occurred in single cases, and one missense variant, c.631G > A (p.Val211Ile), was classified as likely pathogenic. Notably, three patients carried two pathogenic *BRCA2* variants each (Patient 1: c.631G > A and c.7008–2 A > T; Patient 2: c.2557 C > T and c.517-1G > A; Patient 3: c.3860del and c.2600_2601insA). In all three cases, the variants were germline, and it is likely that both variants in each patient are located on the same allele, as both variants in each pair are classified as pathogenic according to ACMG. Compound heterozygosity affecting both *BRCA2* alleles would be incompatible with embryonic viability (Table 3).

**Table 3:** Pathogenic and likely pathogenic variants in *BRCA1* and *BRCA2* genes identified in the course of the study.

№	Gene	hg38 coordinates and variant	HGVS variant name	location	Transcript	*N* of cases
1	*BRCA1*	chr17-43049145-C-CG	c.5381_5382insC (p.Glu1794Aspfs*36)	exon 21	NM_007294.4	12
2	*BRCA1*	chr17-43091462-CTTGA-C	c.4065_4068del (p.Asn1355Lysfs*10)	exon 10	NM_007294.4	1
3	*BRCA1*	chr17-43099850-TAG-T	c.470_471del (p.Ser157*)	exon 7	NM_007294.4	1
4	*BRCA1*	chr17-43057051-C-T	c.5277 + 1G > A	intron 19	NM_007294.4	3
5	*BRCA1*	chr17-43091924-G-A	c.3607 C > T (p.Arg1203*)	exon 10	NM_007294.4	2
6	*BRCA1*	chr17-43092316-AG-A	c.3214del (p.Leu1072*)	exon 10	NM_007294.4	4
7	*BRCA1*	chr17-43094548-CAT-C	c.981_982del (p.Cys328*)	exon 10	NM_007294.4	5
8	*BRCA1*	chr17-43082466-AT-A	c.4294del (p.Ile1432Serfs*2)	exon 12	NM_007294.4	1
9	*BRCA1*	chr17-43094486-C-CG	c.1044_1045ins (p.Glu349Argfs*7)	exon 10	NM_007294.4	1
10	*BRCA1*	chr17-43045792-CTGCCCAAT-C	c.5470_5477del (p.Ile1824Aspfs*3)	exon 23	NM_007294.4	4
11	*BRCA1*	chr17-43106487-A-C	с.181Т> G (p.Cys61Gly)	exon 4	NM_007294.4	1
12	*BRCA2*	chr13-32316484-GCCA-CG	c.24_27delinsCG (p.Arg8Serfs*5)	exon 2	NM_000059.4	2
13	*BRCA2*	--	exon 16 loss	exon 16	NM_000059.4	6
14	*BRCA2*	chr13-32338208-GA-G	c.3860del (p.Asn1287Ilefs*6)	exon 11	NM_000059.4	2
15	*BRCA2*	chr13-32316530-A-G	c.67 + 3 A > G	intron 2	NM_000059.4	1
16	*BRCA2*	chr13-32336955-C-CA	c.2600_2601insA (p.Thr868Tyrfs*13)	exon 11	NM_000059.4	3
17	*BRCA2*	chr13-32380130-G-GA	c.9241_9242insA (p.Val3081Aspfs*30)	exon 24	NM_000059.4	2
18	*BRCA2*	chr13-32338096-TAGTG-T	c.3744_3747del (p.Ser1248Argfs*10)	exon 11	NM_000059.4	1
19	*BRCA2*	chr13-32326613-G-A	c.631G > A (p.Val211Ile)	exon 7	NM_000059.4	1
20	*BRCA2*	chr13-32354859-A-T	c.7008–2 A > T	intron 13	NM_000059.4	1
21	*BRCA2*	chr13-32336912-C-T	c.2557 C > T (p.Gln853*)	exon 11	NM_000059.4	1
22	*BRCA2*	chr13-32326498-G-A	517-1G > A	intron 6	NM_000059.4	1

### Treatment patterns

Treatment differed significantly according to *BRCA* status and tumor subtype (*p* = 0.001). *CDK4/6* inhibitors were administered exclusively in the metastatic *HR* + setting: *BRCA*-negative (*n* = 34), *BRCA2*-positive (*n* = 10), and *BRCA1*-positive (*n* = 1). *PARP* inhibitors were administered exclusively to patients with germline BRCA mutations, including *BRCA1*-positive (*n* = 15) and *BRCA2*-positive (*n* = 2) cases. Among these patients, *PARP* inhibitors were used in both early-stage (*n* = 6) and metastatic (*n* = 9) disease settings. Neoadjuvant chemotherapy was administered mainly to triple-negative patients, including 15 early-stage *BRCA*-negative and 2 metastatic *BRCA*-negative patients. Chemoimmunotherapy was administered to patients with triple-negative breast cancer in both early and metastatic disease settings. In the early-stage breast cancer group, chemoimmunotherapy was provided to 12 *BRCA*-negative, 4 *BRCA1*-positive, and 1 *BRCA2*-positive patients. In the metastatic setting, chemoimmunotherapy was administered to 5 BRCA-negative, 14 *BRCA1*-positive, and 2 *BRCA2*-positive patients. *HER2*-targeted therapy plus chemotherapy was mostly delivered to *HER2*-positive *BRCA*-negative patients (*n* = 28) and a few *BRCA2*-positive metastatic patients (*n* = 2). Adjuvant chemotherapy without neoadjuvant treatment was given to 22 *BRCA*-negative patients. (Table 4) (Supplementary Table 1). The median follow-up time for the entire cohort was 24 months (range 24–72).

**Table 4:** Types of treatment performed depending on *BRCA* status. *ACT* - adjuvant chemotherapy. *NACT* - neoadjuvant chemotherapy. *CDK4/6* - cyclin-dependent kinase 4/6. *PARP* - poly (ADP-ribose) polymerase. *HER2*-human epidermal growth factor receptor 2.

Variables	BRCA-negative *n* = 128 (69%)	BRCA1-positive *n* = 41 (22%)	BRCA2-positive *n* = 17 (9%)	*p* value
Types of treatment				0,001
АCT	22 (17%)	0	0	
NACT	17 (13%)	0	0	
ACT + NACT	1 (1%)	7 (17%)	0	
*CDK4/6* inhibitors	34 (27%)	1 (2%)	10 (59%)	
*PARP* inhibitors	0	15 (37%)	2 (12%)	
Immunotherapy + CТ	17 (13%)	18 (44%)	3 (17%)	
*HER2*neu inhibitors + CТ	28 (22%)	0	2 (12%)	
Surgical treatment	9 (7%)	0	0	

### Survival outcomes

**Fig. 2:**
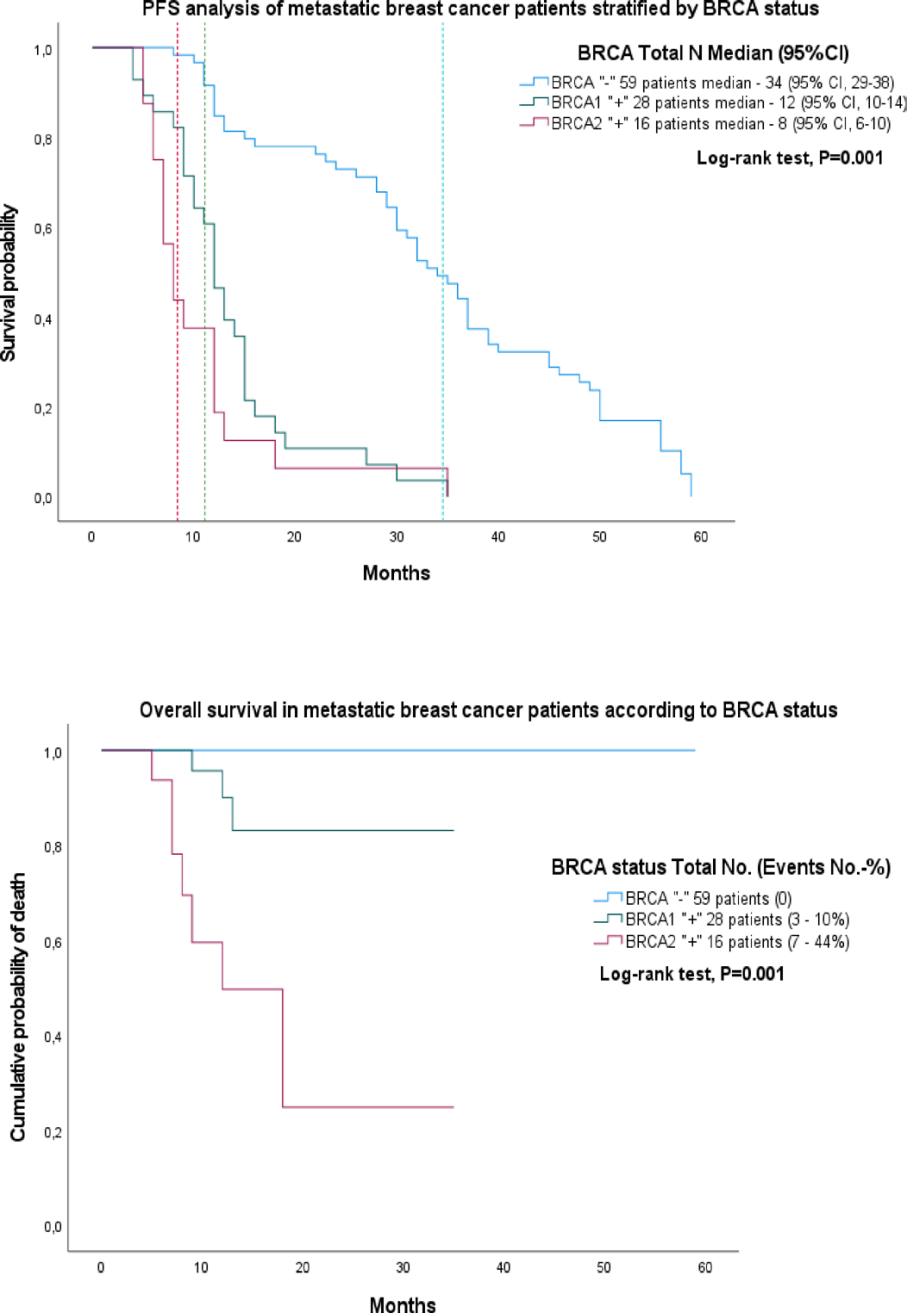
Progression-free and overall survival in metastatic breast cancer according to BRCA status. Kaplan–Meier curves depicting progression-free survival (*2a*-*PFS*, left panel) and overall survival (*2b*-*OS*, right panel) among patients with metastatic breast cancer stratified by *BRCA* status: *BRCA*-negative (*n* = 59), *BRCA1*-positive (*n* = 28), and *BRCA2*-positive (*n* = 16). Pairwise log-rank comparison between BRCA1 and BRCA2 showed no significant difference in PFS (*p* = 0.158) but revealed significantly worse OS for BRCA2 carriers (*p* = 0.001). Overall, both BRCA1- and BRCA2-associated metastatic breast cancers were characterized by poor prognosis, warranting validation in larger cohorts.

Among 103 metastatic breast cancer patients, median progression-free survival (*PFS*) for the metastatic cohort differed significantly by BRCA status (log-rank test, *p* = 0.001): *BRCA*-negative (*n* = 59) 34 months (95% CI, 29–38), *BRCA1*-positive (*n* = 28) 12 months (95% CI, 10–14), and *BRCA2*-positive (*n* = 16) 8 months (95% CI, 6–10) (Fig. 2). Overall survival (OS) differed significantly: no deaths occurred in BRCA-negative metastatic patients; mortality was observed in 3/28 (10%) *BRCA1*-positive and 7/16 (44%) *BRCA2*-positive patients (log-rank test, *p* = 0.001). In pairwise log-rank analyses among metastatic patients, no statistically significant difference in PFS was observed between BRCA1 and BRCA2 carriers (*p* = 0.158), while OS differed significantly, with poorer survival in the BRCA2 subgroup (*p* = 0.001). Overall, carriers of germline BRCA1/2 mutations demonstrated an unfavorable survival profile compared with expectations for unselected metastatic populations; however, these findings should be considered hypothesis-generating and require confirmation in larger, well-powered cohorts (Fig. 2).

**Fig. 3:**
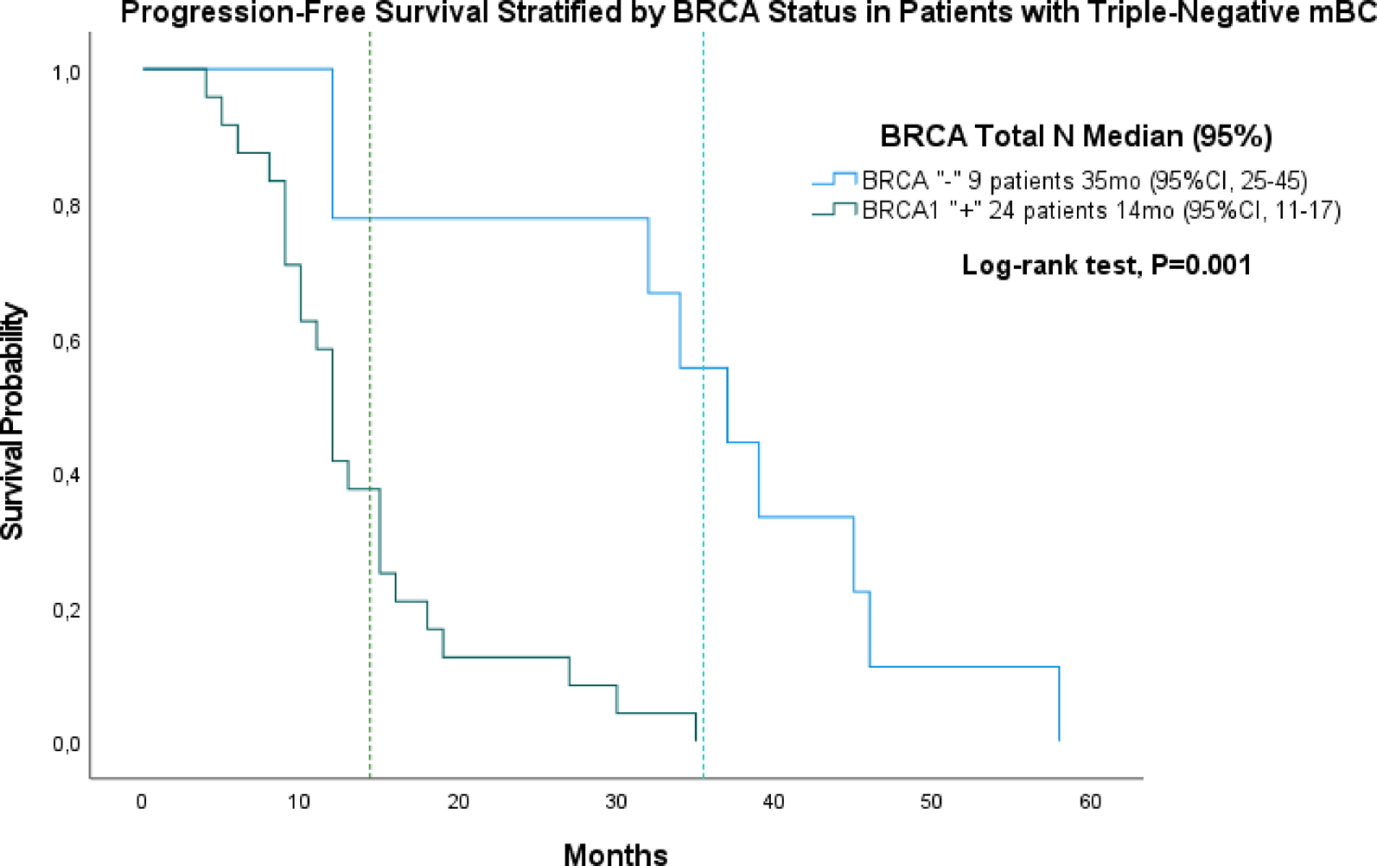
Progression-free survival in triple-negative metastatic breast cancer. Kaplan–Meier curves showing progression-free survival in patients with triple-negative metastatic breast cancer stratified by BRCA status: BRCA-negative (*n* = 9) and *BRCA1*-positive (*n* = 24). *BRCA2*-positive patients were excluded due to insufficient numbers.

In triple-negative metastatic patients (*n* = 33), median *PFS* was 35 months (95% CI, 25–45) for *BRCA*-negative (*n* = 9) and 14 months (95% *CI*, 11–17) for *BRCA1*-positive patients (*n* = 24) (Fig. 3). In *HR* + metastatic patients treated with *CDK4/6* inhibitors (*n* = 43), median *PFS* was 40 months (95% CI, 36–44) in *BRCA*-negative (*n* = 33) and 9 months (95% *CI*, 6–11) in *BRCA2*-positive patients (*n* = 10) (Fig. 3).

**Fig. 4:**
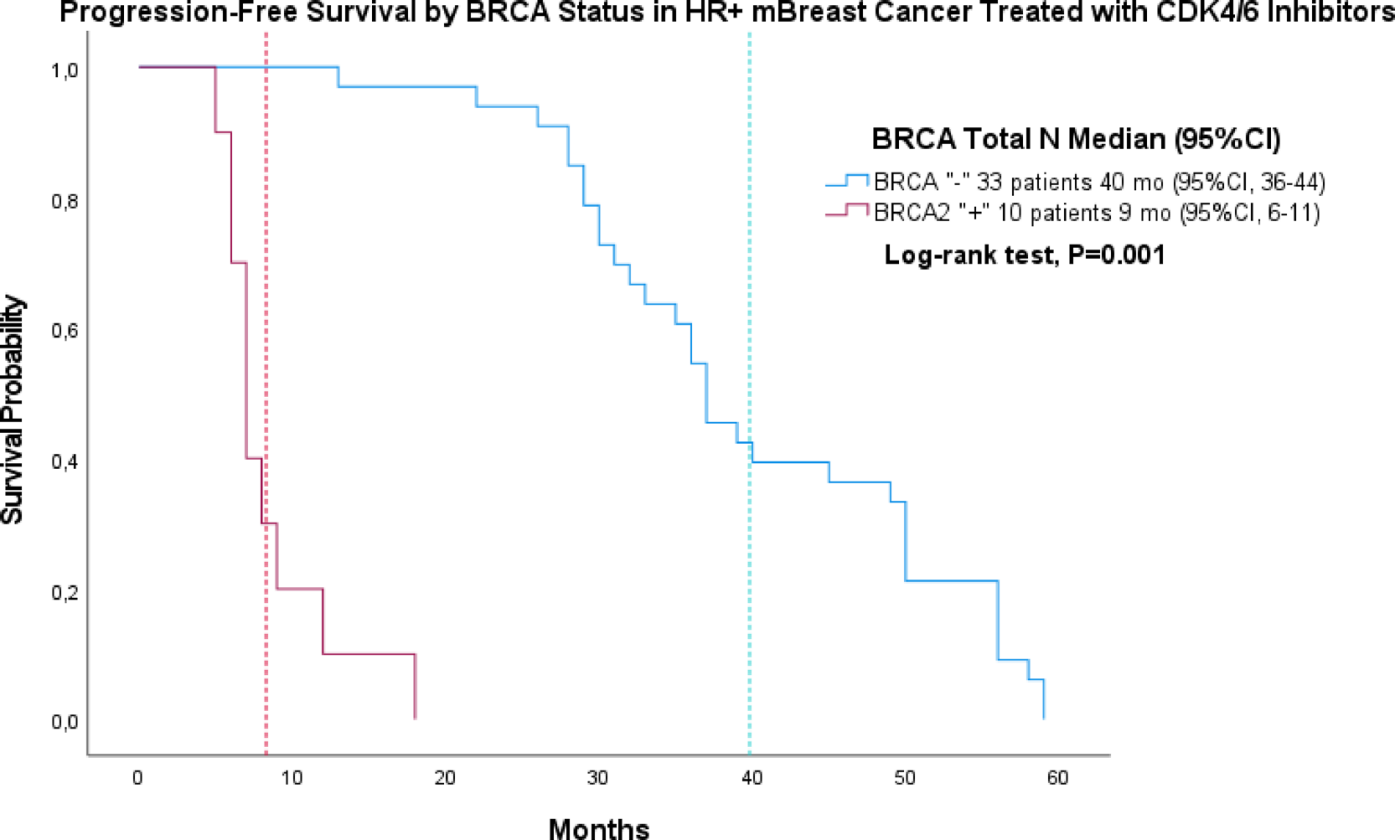
Progression-free survival in HR-positive metastatic breast cancer treated with CDK4/6 inhibitors. Progression-free survival among patients with hormone receptor –positive metastatic breast cancer receiving CDK4/6 inhibitors, stratified by BRCA status: BRCA-negative (*n* = 33) and *BRCA2*-positive (*n* = 10). *BRCA1*-positive patients were not included due to low representation in this treatment subgroup.

## Discussion

### Mutational and clinical profile of *BRCA*-associated breast cancer

Analysis of the mutational profile of breast cancer indicates that abnormalities in *DNA* repair genes, particularly *BRCA1* and *BRCA2* mutations, are critical in the development of aggressive tumor phenotypes and are linked to reduced survival^21^. This research represents the first systematic analysis of *BRCA*-associated breast cancer in Kazakhstan, assessing molecular, morphological, and clinical characteristics, as well as survival differences by *BRCA* status and treatment modality. The findings underscore the importance of investigating *BRCA*-associated breast cancer within the local population to improve prognosis and guide personalized therapeutic strategies.

Among the Kazakhstani cohort, patients with *BRCA1* and *BRCA2* mutations exhibited a significantly lower median age at breast cancer onset compared to those with wild-type *BRCA*, with median ages of 44 years (IQR: 41–48) and 48 years (IQR: 43–54), respectively, versus 51 years (IQR: 46–62) (*p* = 0.001). Additionally, 22% of patients harbored pathogenic variants in *BRCA1*, and 9% harbored pathogenic variants in *BRCA2* (Table 1). This finding indicates that *BRCA*-associated breast cancers develop at a younger age, consistent with multicenter studies from Greece and Japan, highlighting the need for early detection and monitoring in the Kazakhstani population^22,23^. Prospective studies demonstrate that *BRCA1/2* mutation carriers develop breast cancer at a younger age. One cohort reported a median age of 38 years (IQR 30–46) among carriers^24^. While another found the highest risk in *BRCA1* carriers under 35 years, decreasing with age^25^. Similarly, *BRCA1/2* mutations were detected in 15.1% of Moroccan women diagnosed at ≤ 40 years^26^. Together, these findings confirm that *BRCA*-associated breast cancers predominantly arise at a young age, underscoring the importance of early genetic testing, personalized surveillance, and targeted prevention.

Significant differences in disease stage were observed across *BRCA* subgroups (*p* < 0.001). Stages II–III predominated in *BRCA*-negative cases, whereas stages III–IV were more common in *BRCA1*-positive tumors; stage I was absent among *BRCA2* carriers, who mainly presented with stages III–IV (Table 1). These patterns suggest the aggressive nature of *BRCA1*-associated disease and highlight the need for earlier detection in the Kazakhstani population. Similar findings were reported in a Korean cohort, where the frequency of *BRCA1/2* mutations was lower in stage I than in stage 0 and higher in stage II^27^. Most tumors were under 2 cm, with no significant differences in size or lymphovascular invasion among *BRCA* subgroups (*p* = 0.147). Axillary lymph node involvement was more frequent in *BRCA*-negative (61%) and *BRCA2*-positive (71%) patients than in *BRCA1*-positive cases (46%), suggesting distinct invasion patterns (Table 1). The overall high rate of lymphovascular invasion supports the aggressive profile of *BRCA*-related cancers in the Kazakhstani population, consistent with van Barele et al. (2022), who reported increased nodal involvement and reduced 10-year survival with larger tumors^28^.

The identification of recurrent BRCA1/2 pathogenic variants in our cohort is notable from a population genetics perspective. Notably, the BRCA1 c.5266dupC (5382insC) variant, the most frequently observed alteration, is a known founder mutation in several Eastern European populations, including Poland^29^, suggesting possible shared ancestral origins or regional mutation patterns. However, our study was not designed to formally assess founder effects. These variants are therefore reported as recurrent pathogenic alterations rather than confirmed founder mutations. Future large-scale studies incorporating haplotype analysis and representative population controls will be necessary to clarify founder contributions in Kazakhstan (Table 3).

Metastatic patterns differed across *BRCA* subgroups. Absence of metastases was observed in 54% of *BRCA*-negative, 32% of *BRCA1*, and 6% of *BRCA2* carriers (*p* = 0.001). *BRCA1* tumors more often spread to the lungs and distant lymph nodes, whereas *BRCA2* tumors showed multiple bone and multi-organ metastases (Table 1). Similar trends have been reported previously, with predominant lung and nodal metastases in *BRCA1* and bone involvement in *BRCA2* and *BRCA*-negative cases (*p* = 0.010)^30^. Multiple primary cancers, mainly breast–ovarian combinations, were frequent among *BRCA1* carriers^31^. Moreover, a meta-analysis found higher mean *OncotypeDX* scores in *BRCA*-mutated versus sporadic cases (25 vs. 18.4; *p* < 0.001), indicating a more aggressive molecular phenotype^32^. These findings emphasize the need for early risk stratification and population-specific research in Kazakhstan.

The pathological profile showed notable differences between subgroups. Moderately differentiated tumors (*G2*, 45%) were more common in the *BRCA*-negative group, while poorly differentiated tumors (*G3*, 61% and 53%; *p* = 0.002) were more frequent in the *BRCA*-positive group (Table 2). A retrospective study by Krammer et al. (2017) reported similar findings, with most *BRCA1*-associated tumors classified as *G3* and having a higher histological grade than *BRCA2* (*p* < 0.001)^33^. The median *Ki-67* proliferation index was 40% (IQR: 20–55) and was significantly higher in *BRCA1*-positive patients compared to *BRCA*-negative patients (63% vs. 16%, *p* = 0.001), indicating increased cellular proliferation and more aggressive tumor behavior. Furthermore, *BRCA1*-associated tumors exhibited higher proliferative activity than *BRCA2*-associated tumors, with median Ki-67 values of 75% and 55%, respectively (Table 2), supporting the association of *BRCA1* mutations with a more aggressive tumor phenotype^34^.

In the Kazakhstani population, *BRCA1*-associated tumors were predominantly triple-negative (88%), compared with 29% of *BRCA2* and 12% of *BRCA*-negative cases (*p* = 0.001) (Table 2), reflecting disrupted hormonal signaling and endocrine resistance. Owing to defects in homologous recombination, these tumors remain sensitive to platinum-based agents and *PARP* inhibitors^35^. In our cohort, 88% of *BRCA1*-positive patients had triple-negative tumors, compared to 29% of *BRCA2* carriers and 12% of *BRCA*-negative patients (*p* = 0.001) (Table 2).

Similar findings were reported in a Japanese cohort, where 75.8% of *BRCA1*-associated tumors were triple-negative^36^. *BRCA2*-associated tumors showed hormone receptor positivity (*ER/PR*, 65%; *p* = 0.001), moderate differentiation, and a low *Ki-67* index, consistent with luminal phenotypes and favorable clinical behavior. Guzmán-Arocho et al. (2022) reported comparable results, with 32.9% luminal A-like and 55.3% luminal B-like *BRCA2*-positive tumors^37^. In our sample, 65% of *BRCA2*-positive patients were *ER*- and *PR*-positive (*p* = 0.001) (Table 2). Despite these features, BRCA2 carriers in our cohort exhibited a trend toward shorter progression-free and overall survival, underscoring the need for tailored therapeutic approaches in the Kazakhstani population; however, this observation should be interpreted with caution and requires confirmation in larger, well-powered cohorts.

In Poland, systematic *BRCA1*/2 screening has been implemented over the past two decades, with a focus on three founder mutations (5382insC, C61G, and 4153delA). This program, conducted at the Pomeranian Medical University in collaboration with the Women’s College Research Institute, University of Toronto, has enrolled thousands of carriers and provided robust prospective data on hereditary breast and ovarian cancer^29^. The experience demonstrates how population-specific genetic programs can facilitate early detection and preventive strategies, offering a model that may inform the development of similar initiatives in other countries, including multiethnic populations like Kazakhstan.

Our analysis revealed distinct molecular and clinical features of *BRCA*-associated breast cancer, marked by earlier onset, poor differentiation, triple-negative phenotype, high proliferative activity, and frequent visceral metastases, indicating a more aggressive course than sporadic cases. These findings emphasize the role of *DNA* repair defects in reduced survival and highlight the need for early molecular testing and personalized treatment in Kazakhstan.

### Molecular heterogeneity and prognostic significance of *BRCA* mutations in different subtypes of breast cancer

In the overall survival analysis, statistically significant differences were observed between BRCA2-positive patients and both BRCA1-positive and BRCA-negative groups, with BRCA2 carriers demonstrating inferior outcomes. In contrast, no statistically significant difference in overall survival was detected between BRCA1-positive and BRCA-negative patients (log-rank *p* = 0.40). Notably, a numerically more favorable survival trend was observed in the BRCA1-positive subgroup compared with BRCA2-positive patients. Given the limited sample size and the number of observed events, these findings should be considered hypothesis-generating. Further studies in larger, independent cohorts with sufficient statistical power are required to clarify potential differences in overall survival between BRCA1-positive and BRCA-negative patients and to confirm the observed survival gradient across BRCA subgroups.

The prognostic value of germline *BRCA* mutations in breast cancer remains controversial. Among metastatic patients, *BRCA1* carriers exhibited a median progression-free survival *(PFS)* of 12 months, while *BRCA2* carriers had a shorter *PFS* of 8 months, compared with 34 months in *BRCA*-negative patients. Overall survival (*OS*) was markedly reduced in *BRCA2*-positive patients, whereas no deaths were observed in *BRCA*-negative metastatic patients (*p* = 0.001) (Fig. 2). These findings contrast with the prospective POSH study (Copson et al., 2018), which reported no survival difference between *BRCA*-positive and *BRCA*-negative patients (HR = 0.96; 95% CI: 0.76–1.22; *p* = 0.76)^38^. While Schmidt et al. (2017) observed worse early survival in *BRCA1* and later in *BRCA2* carriers^39^, our data indicate earlier progression and poorer outcomes in *BRCA2*-associated tumors, likely reflecting population-specific factors, tumor biology, and access to modern therapy.

These findings are partly consistent with Liu et al. (2021), who analyzed 30 studies including 35,972 patients (mean age 45.6 years) and reported reduced OS in *BRCA1* carriers (HR = 1.20; 95% *CI*: 1.08–1.33; *p* < 0.001), lower *PFS* (HR = 1.35; 95% *CI*: 1.10–1.67; *p* = 0.0049), and decreased breast cancer–specific survival in *BRCA2* carriers (HR = 1.46; 95% CI: 1.26–1.70; *p* < 0.0001). In triple-negative breast cancer, *BRCA1* mutations further worsened *PFS* (HR = 1.65; 95% CI: 1.08–2.54; *p* = 0.0216)^21^. Similarly, Feszak et al. (2025) showed that the prognostic effect of *BRCA* mutations varies by tumor subtype, with *BRCA1/2* carriers with *DCIS* demonstrating favorable outcomes over 110 months. Consistent with these observations, in our cohort of triple-negative metastatic breast cancer patients, BRCA1-positive individuals exhibited shorter PFS compared with BRCA-negative patients. Although limited by small sample size, these findings support the notion that BRCA1-associated triple-negative tumors display more aggressive clinical behavior (Fig. 3). Together, these data highlight the biological heterogeneity of *BRCA*-associated tumors and suggest that poorer *PFS* and OS in *BRCA2* carriers may reflect more aggressive invasive disease or therapy response differences^40^.

This study, among the first in Kazakhstan, evaluated the impact of *BRCA* status on survival in metastatic breast cancer, accounting for hormonal profiles. *BRCA*-associated tumors, particularly *BRCA2*-mutated, exhibited more aggressive behavior, possibly reflecting population-specific and biological factors. Among metastatic *HR*⁺ breast cancer patients, our results showed significant variation by *BRCA* status: *BRCA*-negative patients had the longest *PFS* (median: 40 months; *p* = 0.001), whereas *BRCA2* carriers progressed earlier (median: 9 months), likely due to impaired *DNA* repair and reduced endocrine sensitivity, despite receiving *CDK4/6* inhibitors (Fig. 4). Similarly, a meta-analysis of 22 studies (*n* = 34,960) found poorer relapse-free (HR = 1.64; 95% CI: 1.00–2.69; *p* = 0.05) and overall survival (HR = 1.52; 95% CI: 1.20–1.92; *p* = 0.0006) in *HR*-positive patients with *BRCA* mutations. These data align with Azim et al. (2025), who linked RB1 loss of heterozygosity in *BRCA2*-positive tumors to endocrine and *CDK4/6* inhibitor resistance^41^.

In recent studies, both *BRCA* mutation type and tumor hormonal status have been shown to influence breast cancer outcomes. Lambertini et al. (2021) (*n* = 1236) reported that *BRCA1* carriers more often developed *HR*-negative tumors and second malignancies, while *BRCA2* mutations were associated with poorer relapse-free survival (adjusted *HR* = 0.76; 95% CI: 0.60–0.96)^42^. Similarly, our data revealed worse prognosis among *HR*⁺ *BRCA2* carriers (*p* = 0.001). Vocka et al. (2019) found that *HR*⁺ patients with *BRCA* mutations had higher recurrence (38.3% vs. 16.6%; *p* < 0.001) and mortality (21.7% vs. 6.3%; *p* < 0.001), confirming the aggressive behavior of hormone-dependent subtypes^43^.

Consistent with these findings, a real-world observational analysis from the French *ESME* metastatic breast cancer platform evaluated the prognostic impact of germline *BRCA1/2* mutations specifically in *HR*^+^/*HER2*^-^ metastatic breast cancer. Among 13,776 patients, 170 were germline BRCA1/2 mutations (*gBRCAm)* carriers, 676 had *BRCA* wild-type tumors, and 12,930 were untested. Multivariable time-dependent analyses demonstrated inferior overall survival in *gBRCAm* carriers compared with *BRCA* wild-type patients (adjusted *HR* 1.26; 95% *CI*: 1.03–1.55). In the subgroup receiving frontline endocrine therapy, *gBRCAm* status was associated with significantly worse overall survival (adjusted HR 1.54; 95% *CI*: 1.03–2.32) and first-line progression-free survival (adjusted *HR* 1.58; 95% *CI*: 1.17–2.12). These data corroborate the detrimental prognostic impact of *BRCA* mutations in *HR*^+^ metastatic disease and underscore the need for genotype-guided therapeutic strategies^44^.

In our cohort, metastases were more frequent in *BRCA1/2* carriers (*p* = 0.001): *BRCA1* was associated with lung and genital tract involvement, whereas *BRCA2* was associated with multi-organ spread. The *ESME* study (Mailliez et al., 2023) similarly reported increased visceral metastases and a more aggressive course in *BRCA*-positive patients, with shorter OS but no independent prognostic effect. Notably, *TNBC* patients with *BRCA* mutations had better OS and *PFS*1, while *HR*⁺/*HER2*⁻ carriers had worse outcomes^17^, highlighting the prognostic relevance of molecular subtype and metastatic pattern.

Our study in a Kazakhstani cohort demonstrates that population-specific and molecular factors influence the prognostic impact of *BRCA* mutations in *HR*⁺ breast cancer. The reduced *PFS* and OS observed in *BRCA2* carriers may reflect genetic heterogeneity and differences in treatment. These findings highlight the need for routine *BRCA* testing in *HR*⁺ metastatic breast cancer and support personalized therapeutic strategies integrating endocrine therapy, *CDK4/6*, and *PARP* inhibitors.

### Impact of *BRCA1* and *BRCA2* mutations on clinical outcomes and therapeutic effectiveness

The study revealed distinct clinical and therapeutic differences between *BRCA1* and *BRCA2* mutation carriers, reflecting the biological heterogeneity of *BRCA*-associated breast cancer. Nowadays, *BRCA*-positive patients are preferentially treated with *PARP* inhibitors, reflecting enhanced sensitivity of *BRCA*-deficient tumors to *DNA*-damaging agents^45^. Among our cohort, *PARP* inhibitors were administered in both early-stage (*n* = 6) and metastatic (*n* = 9) settings. Neoadjuvant chemotherapy was mainly given to triple-negative patients (15 early-stage and 2 metastatic *BRCA*-negative); however, due to the small cohort size, survival outcomes could not be reliably assessed (Supplementary Table 1). Given the small number of *HER2*-positive cases among *BRCA*-positive patients, particularly the absence of *BRCA1/HER2*-positive tumors, treatment-specific prognostic conclusions, including response to neoadjuvant chemotherapy and anti-*HER2* therapy, could not be sufficiently evaluated and require validation in larger cohorts. Phase III trials demonstrated the superiority of *PARP* inhibitors over standard chemotherapy in *BRCA*-mutated, *HER2*-negative metastatic breast cancer. Olaparib improved median progression-free survival (7.0 vs. 4.2 months; *HR* = 0.58; *p* < 0.001) and response rate (59.9% vs. 28.8%), while talazoparib showed similar benefits (8.6 vs. 5.6 months; *HR* = 0.54; *p* < 0.001; responses 62.6% vs. 27.2%), with both agents offering better tolerability^46^.

In the triple-negative subgroup, progression-free survival significantly differed by *BRCA* status (Log-rank test, *p* = 0.001) (Fig. 3): *BRCA*-negative patients (*n* = 9) had a median *PFS* > 30 months, whereas *BRCA1* (*n* = 24) carriers showed accelerated progression within 10–15 months. These findings are consistent with those of Copson et al. (2018) and support the enhanced short-term responsiveness of *BRCA*-mutated *TNBC* to *DNA*-damaging and platinum-based therapies^38^.

*BRCA*-positive patients in our cohort received targeted therapies reflecting tumor biology, including *PARP* inhibitors in early-stage (*n* = 6) and metastatic (*n* = 9) settings, as well as chemoimmunotherapy for triple-negative disease. Neoadjuvant chemoimmunotherapy was given to 15 early-stage and 2 metastatic *BRCA*-negative patients, and to *BRCA1/2* carriers in both settings; however, survival outcomes could not be reliably assessed due to the small cohort size (Supplementary Table 1). Although the small cohort size precluded reliable assessment of survival outcomes, these treatment patterns align with previous evidence demonstrating the benefit of combining immunotherapy with chemotherapy. For instance, Schmid et al. (2020) reported that adding pembrolizumab to neoadjuvant chemotherapy in early *TNBC* improved pathological complete response (64.8% vs. 51.2%; *p* < 0.001) and reduced the risk of progression or death (HR = 0.63; 95% *CI*: 0.43–0.93), highlighting the potential synergistic interaction between immunotherapy and *DNA* repair-targeted strategies^47^. *PARP* inhibitors demonstrate strong antitumor potential in this group and warrant further study to define their optimal integration into treatment^48^.

Patients with *BRCA*-negative status receiving *CDK4/6* inhibitors had a markedly longer progression-free interval during the first 40 months compared with *BRCA*-mutated patients with metastatic breast cancer. This observation aligns with findings from a meta-analysis including 618 *gBRCAm* patients with *HR+/HER2 −* metastatic breast cancer, in which *gBRCAm* status was consistently associated with inferior outcomes on *CDK4/6* inhibitors. Specifically, *gBRCAm* carriers experienced significantly shorter *PFS* (*HR* 1.68; 95% *CI* 1.37–2.05) and *OS* (*HR* 1.73; 95% *CI* 1.12–2.67) compared with *gBRCAwt* patients. Collectively, these data suggest a clinically meaningful reduction in *CDK4/6* inhibitor efficacy in *gBRCAm* tumors, possibly related to dysregulated *DNA* damage response pathways and altered cell-cycle control^49^.

Our findings demonstrate that *BRCA1*/2 mutations shape tumor biology and treatment response, emphasizing the need for genotype-guided therapy. In the Kazakhstani population, which is multiethnic, *BRCA2*-positive patients exhibited a more aggressive disease course, likely reflecting molecular and population-specific differences. Due to the high cost of genetic testing and limited resources, the study included all eligible patients rather than a homogeneous cohort, providing a realistic representation of clinical practice. Consistent with global evidence, these results support routine *BRCA* testing for *HR* + and *TNBC* subtypes and the implementation of personalized regimens integrating *PARP* inhibitors, selective *CDK4/6* blockade, and immunotherapy. As the first comprehensive analysis of *BRCA*-associated breast cancer in Kazakhstan, this study highlights disparities in access to genetic testing and targeted therapies, particularly in rural regions. Strengthening national programs for genetic counseling and expanding access to precision treatments are essential to improving survival outcomes and ensuring equitable cancer care.

## Conclusion

In conclusion, our findings emphasize the clinical and prognostic importance of germline *BRCA* testing in breast cancer management. *BRCA1*-positive tumors were characterized by earlier onset, high-grade histology, elevated proliferative activity, and a predominance of triple-negative subtypes, underscoring the need for tailored therapeutic strategies. *BRCA2*-associated cancers, although frequently hormone receptor–positive, presented with advanced disease and inferior survival, indicating the potential benefit of early systemic interventions and inclusion in trials evaluating novel targeted therapies. The observed patterns confirm the biological heterogeneity of *BRCA*-driven breast cancer and align with international data, suggesting the absence of major region-specific modifier genes. These results highlight the value of integrating molecular diagnostics into clinical practice to identify high-risk patients for genotype-guided interventions, including *PARP* inhibitors, and support the implementation of nationwide genetic screening and counseling programs. Overall, personalized treatment strategies that account for genetic background, tumor characteristics, and healthcare system factors are crucial for improving outcomes and advancing precision oncology in Kazakhstan.

## Limitations

The ambispective design and the relatively small, single-center cohort may limit the generalizability of the findings. Also, the study was not designed to assess founder effects, and the absence of haplotype analysis or population-based reference data limits interpretation of recurrent variants in the Kazakhstani population. Data on subsequent lines of therapy, including novel targeted agents and participation in clinical trials, were incomplete. Additionally, the lack of comprehensive genomic profiling beyond *BRCA1/2* mutations limits our ability to fully characterize co-occurring mutations that may influence prognosis and therapy response. The restricted availability and high cost of *BRCA* testing further constrained broader genetic screening, and both metastatic and non-metastatic patients were analyzed together due to time limitations. Finally, follow-up duration varied across patients, potentially affecting long-term survival estimates.

## Future directions

Future research should focus on multicenter prospective studies to validate these findings and assess long-term outcomes. Expanding genomic profiling beyond *BRCA1/2* to include other homologous recombination repair genes may improve risk stratification and treatment selection. Clinical trials evaluating the efficacy and safety of *PARP* inhibitors, *CDK4/6* inhibitors, and immunotherapy in young *BRCA1/2* carriers are urgently needed in Kazakhstan. Integration of regional genetic data with treatment response and survival outcomes will enable the development of personalized, evidence-based protocols tailored to the Central Asian population.

## Supplementary Information

Below is the link to the electronic supplementary material.

Supplementary Material 1

## Data Availability

All data generated or analyzed during this study are available from the corresponding author on reasonable request.

## Abbreviations

BRCA1/2: breast cancer gene 1 and 2
NGS: next-generation sequencing
IHC: immunohistochemistry
FISH: fluorescence in situ hybridization
HR: hormone receptor
ER/PR: estrogen/ progesterone receptor
HER2neu: Human epidermal growth factor receptor 2
G: tumor grade
Ki-67: proliferation marker
CI: confidence interval
DFS/PFS: disease-/progression free survival
OS: overall survival
CDK4/6: cyclin-dependent kinase 4 and 6
PARP: poly (ADP-ribose) polymerase
NACT: neoadjuvant chemotherapy
ACT: adjuvant chemotherapy
TNBC: triple-negative breast cancer
DCIS: Ductal Carcinoma in Situ
