# Supplementary material for: Molecular characteristics and prognostic insights into BRCA-associated breast cancer in Kazakhstan
Source: Sci Rep. 2026 Jan 17;16:5652. doi: 10.1038/s41598-026-36086-0 (PMC12891505; doi:10.1038/s41598-026-36086-0)
Supplement: Supplementary file 1 — Supplementary Material 1 [file 41598_2026_36086_MOESM1_ESM.pdf]

Supplementary Information for: «Molecular Characteristics and Prognostic Insights into BRCA-Associated Breast Cancer in Kazakhstan».

Authors: Ainur Samigatova[1*], Nursulu Altaeva[2], Yerlan Suleimenov[3], Petr Sibiryakov[4], Kuantkan Zhabagin[5], Zhanar Zhakypbekkyzy[6], Bakhytzhan Seksenbaev[7], Noso Yoshihiro[8], Oxana Tsigengagel[9]

Affiliations:

[1*]Astana Medical University, QazGene-LLP, Astana, Kazakhstan;
[2]Astana Medical University, Department of Medical Genetics and Molecular Biology, Astana, Kazakhstan;
[3]CDL OLYMP- LLP, QazGene-LLP, Astana, Kazakhstan;
[4]GeneNote LLP, Astana, Kazakhstan;
[5]Medical Center hospital of the president's affairs administration of the republic of Kazakhstan, Astana, Kazakhstan;
[6]Astana Medical University, Astana, Kazakhstan;
[7]Astana Medical University, Astana, Kazakhstan;
[8]Hiroshima International University, Kurose gakuendai, Higashi hiroshima, Hiroshima, Japan;
[9]Astana Medical University, Astana, Kazakhstan.

ORCID

Ainur Samigatova https://orcid.org/0000-0002-2919-8212

Nursulu Altaeva https://orcid.org/0000-0002-8756-1462

Yerlan Suleimenov https://orcid.org/0009-0003-2629-7164

Petr Sibiryakov https://orcid.org/0000-0002-2552-0323

Kuantkan Zhabagin https://orcid.org/0000-0002-4304-5132

Zhanar Zhakypbekkyzy https://orcid.org/0009-0009-4943-1980

Bakhytzhan Seksenbaev https://orcid.org/0009-0004-4931-6935

Noso Yoshihiro https://orcid.org/0000-0003-3477-1260

Oxana Tsigengagel https://orcid.org/0000-0002-3170-9712

***Address for Correspondence**:

Ainur Samigatova, M.D., Ph.D. candidate
Astana Medical University, QazGene LLP, Astana, Kazakhstan, Beibitshilik Street 49/A, Astana 010000, Kazakhstan
Email: ainur.samigatova86@gmail.com
Phone number: +77025460313

Supplementary Table 1. Treatment patterns according to disease stage, molecular subtype, and BRCA status

	0-*ACT*	1-*NACT*	2-*NACT/ ACT/CT*	3-*CDK4/6* inhibitors	4-*PARP* inhibitors	5-Immunotherapy+CT	6-*HER2*neu inhibitors+CT	7-Surgical treatment	Total
**All patients with BC, n=186**									
BRCA negative	22	17	1	34	-	17	28	9	128
BRCA1 positive	-	-	7	1	15	18	-	-	41
BRCA2 positive	-	-	-	10	2	3	2	-	17
**Patients with early BC, n=83**									
BRCA negative	22	15	-	-	-	12	11	9	69
BRCA1 positive	-	-	3	-	6	4	-	-	13
BRCA2 positive	-	-	-	-	-	1	-	-	1
**Patients with metastatic BC, n=103**									
BRCA negative	-	2	1	34	-	5	17	-	59
BRCA1 positive	-	-	4	1	9	14	-	-	28
BRCA2 positive	-	-	-	10	2	2	2	-	16
**Triple negative, total, n=63**									
BRCA negative	1	2	1	1	-	17	-	-	22
BRCA1 positive	-	-	6	-	15	15	-	-	36
BRCA2 positive	-	-	-	-	2	3	-	-	5
**Triple negative early breast cancer, n=26**									
BRCA negative	1	-	-	-	-	12	-	-	13
BRCA1 positive	0	-	2	-	6	4	-	-	12
BRCA2 positive	-	-	-	-	-	1	-	-	1
**Triple-negative metastatic breast cancer, n=37**									
BRCA negative	-	2	1	1	-	5	-	-	9
BRCA1 positive	-	-	4	-	9	11	-	-	24
BRCA2 positive	-	-	-	-	2	2	-	-	4

**Hormone receptor positive breast cancer, total, n=118**									
BRCA negative	21	15	-	33	-	-	24	9	102
BRCA1 positive	-	-	1	1	-	3	-	-	5
BRCA2 positive	-	-	-	10	-	-	1	-	11
**Hormone receptor positive breast cancer (early cancer), n=56**									
BRCA negative	21	15	-	-	-	-	10	9	55
BRCA1 positive	-	-	1	-	-	-	-	-	1
BRCA2 positive	-	-	-	-	-	-	-	-	-
**Hormone receptor positive breast cancer (metastatic cancer), n=62**									
BRCA negative	-	-	-	33	-	-	14	-	47
BRCA1 positive	-	-	-	1	-	3	-	-	4
BRCA2 positive	-	-	-	10	-	-	1	-	11
**HER2 positive all breast cancer patients, n=31**									
BRCA negative	-	-	-	-	-	-	27	1	28
BRCA1 positive	-	-	-	-	-	-	-	-	-
BRCA2 positive	-	-	-	1	-	-	2	0	3
**HER2 positive all breast cancer patients (early cancer), n=11**									
BRCA negative	-	-	-	-	-	-	10	1	11
BRCA1 positive	-	-	-	-	-	-	-	-	-
BRCA2 positive	-	-	-	-	-	-	-	-	-
**HER2 positive all breast cancer patients (metastatic cancer), n=20**									
BRCA negative	-	-	-	-	-	-	17	-	17
BRCA1 positive	-	-	-	-	-	-	-	-	
BRCA2 positive	-	-	-	1	-	-	2	-	3
